# Lighting and circadian cues shape locomotor strategies for balance and navigation in larval zebrafish

**DOI:** 10.1101/2025.11.18.689084

**Published:** 2025-11-18

**Authors:** Jiahuan Liu, Samantha N. Davis, Hannah Gelnaw, David Schoppik, Yunlu Zhu

**Affiliations:** 1 Departments of Neuroscience, Otolaryngology, and the Institute for Translational Neuroscience, NYU Grossman School of Medicine, USA; 2 Department of Psychology, New York University, USA; 3 Lead Contact

## Abstract

Most fish are inherently unstable and must swim to stabilize posture. How diurnal fish reduce activity at night while maintaining postural control remains unclear. We defined distinct locomotor strategies that larval zebrafish (*Danio rerio*) use to control posture and navigate the water column in response to light and circadian cues. In the dark, larvae maintain balance by swimming in long bouts with large nose-up rotations, compensating for nose-down drift accrued during prolonged inactivity. Effective postural compensation requires vestibular sensation from the utricle. By contrast, in the light, larvae navigate with short, frequent, and variable bouts. While lighting exerts a dominant, masking effect on the locomotor strategies, circadian rhythms modulate the extent of each strategy. Our results reveal distinct day-night locomotor strategies and disentangle how ambient light and the internal clock jointly shape balance control and navigation. This work lays the foundation for understanding how external and internal cues interact to govern locomotor activity in freely moving diurnal animals.

## INTRODUCTION

Environmental lighting and the internal circadian clock interact to regulate most animals’ locomotor activity. The internal clock drives rhythmic behavior [1, 2], while light acts both as a dominant zeitgeber that entrains the clock [3, 4] and as a direct modulator of behavior through its masking effects [5–8]. Terrestrial animals that are active during the day rest at night by remaining still [9–11]. However, zebrafish (*Danio rerio*), like most fish species, lack a stable body plane [12, 13], requiring active swimming to maintain their preferred horizontal posture [14]. How diurnal fish stabilize posture despite reduced locomotor activity at night remains unknown.

Larval zebrafish are an ideal system for studying how lighting and circadian rhythms shape locomotor behavior. They are more active during the day [15–22] and display light-induced masking of activity [23, 24]. Zebrafish larvae swim in discrete bouts separated by periods of inactivity [25, 26], making their locomotion easy to quantify. In the first few days post fertilization (dpf), larvae explore the water column to inflate their swim bladder at the surface [27, 28], hunt microorganisms [29], and adjust depth in response to illumination changes [23, 30, 31]. Zebrafish larvae experience a constant nose-down torque due to their front-heavy body plane [13, 14]. To maintain a preferred horizontal posture and navigate the water column effectively, they must sense and correct body tilts along the pitch (nose-up/nose-down) axis using vestibular feedback [14, 26, 32]. Although lighting and circadian cues influence activity levels in zebrafish larvae, their impact on the kinematics governing postural control and navigation remains unexplored.

We combined high-resolution behavioral recordings with photoperiodic manipulations to dissect how lighting and circadian rhythms shape locomotor strategies in larval zebrafish. We found that larvae adopt two distinct strategies depending on light availability and identified bout duration as a key kinematic parameter that classifies these two strategies. In the dark, larvae prioritized postural stabilization though long swim bouts accompanied by pronounced nose-up rotations. These rotations effectively compensated for nose-down postural drifts accrued during prolonged inactivity. In the light, larvae adopted frequent, short swim bouts with high directional variability, facilitating horizontal exploration. Transitions between lighting conditions induced quick strategy switching. Interestingly, while lighting exerts a dominant masking effect on locomotor strategies, circadian rhythms modulate the extent of each strategy. Postural control and navigation exhibited rhythmic fluctuations under both constant light and constant darkness. In conclusion, our study disentangles the contributions of lighting and circadian rhythms to locomotor activity, revealing how a simple vertebrate adjusts locomotor strategies to balance and navigate in response to external and internal cues.

## MATERIALS AND METHODS

### Fish husbandry

All procedures involving larval zebrafish (*Danio rerio*) have been approved by the Institutional Animal Care and Use Committee (IACUC) at New York University Langone Health. Adult zebrafish were maintained at 28.5 °C under a standard 14/10-hour light/dark cycle with lights on from 9 a.m. to 11 p.m. Fertilized embryos were derived from in-crosses of wild-type zebrafish with a mix of AB/WIK/TU/SAT/NHGRI-1 backgrounds. During the first day after birth, embryos were raised at densities ranging from 20 to 50 in 10 cm petri dishes, each containing 25 to 40 mL of E3 medium with 0.5 ppm methylene blue. At 1 day post-fertilization (dpf), larvae were transferred to E3 medium without methylene blue. Starting from 5 dpf, larvae were raised at a density around 20 per dish and fed with cultured rotifers (Reed Mariculture) daily. Larvae were kept in incubators set to a 28.5 °C 14/10-hour light/dark cycle until behavioral assessment starting at 7 dpf.

### Behavioral measurements

We used the Scalable Apparatus to Measure Posture and Locomotion (SAMPL) to record behavior of freely swimming zebrafish larvae [25]. The apparatus, experimental procedure, program, and analysis pipeline have been detailed previously [25]. Briefly, at 7 dpf, 5 to 7 larvae were transferred into each custom-designed behavior chamber filled with 30 mL of E3 medium. The water filled a portion of the chamber that measured approximately 50 mm (L) × 50 mm (H) × 13 mm (W). Each chamber was positioned between a camera and a 940 nm infrared light source inside a light-tight box. Each behavior box was equipped with a cool white LED strip to control photoperiodic treatments, illuminating the chamber with cool white light measured between 50 to 150 lux. Behavioral recordings began before 11 a.m. on the first day and lasted for approximately 48 hours. The recording field of view was a 20 mm × 20 mm square in the center of the lower half of the chamber. We recorded the coordinates and angle along the pitch axis (nose-up/nose-down) of larvae in the field of view in real time at 166 Hz. After approximately 24 hours, recordings were paused for 30 minutes, where each chamber was fed with 1 to 2 mL of cultured rotifers.

Constant light (LL) and constant dark (DD) conditions were generated by keeping the white LED on or off, respectively. For the light/dark (LD) condition, the white LED followed a standard 14/10-hour light/dark cycle with the light on from 9 a.m. to 11 p.m. Each experimental repeat included data from all three photoperiodic treatments, collected from larvae of the same clutch (siblings). Each condition per repeat contained data from three SAMPL boxes with approximately 20 fish combined. Refer to figure legends and tables for the exact number of experimental repeats, number of fish, and number of swim bouts.

### Lesions of lateral line hair cells

At 7 dpf, larvae were treated with 10 μM copper sulfate (CuSO_4_) in E3 medium for 90 minutes. Control siblings were handled identically and transferred to E3. After the treatment, larvae were washed in E3 and transferred to behavioral chambers. Following 24 hours of behavioral assessment, the CuSO_4_ treatment was repeated to prevent hair cell regeneration. Quantification of lateral line hair cell loss following the CuSO_4_ treatment can be found in [33].

### Behavioral analysis

Raw behavioral data were preprocessed using pipelines previously published with the SAMPL apparatus [25]. First, swim speed was calculated from recorded coordinates. A speed threshold of 5 mm/s was used to detect swim bouts. Then, we segmented the time-series data into swim bouts and inter-bout intervals (IBIs) by extracting a 450 ms window around the time of peak speed for each bout. Swim bouts were then aligned at the time of peak speed for subsequent analyses.

Next, we analyzed the preprocessed data to extract kinematic parameters. For each bout, we calculated the peak speed, bout duration, swim displacement, direction, and body rotation. To capture the temporal dynamics of swim speed, we defined the bout duration as the duration of time that swim speed remained above 50% of its peak, i.e. the width at half maximum of the speed profile. Swim displacement was determined as the Euclidean distance traveled during the period when speed remained faster than 5 mm/s. Swim direction was defined as the instantaneous trajectory at the time of the peak speed on the pitch (nose-up/nose-down) axis. To assess rotation, we analyzed the body angle along the pitch axis relative to horizontal. Bout rotation was defined as the net change in pitch angle between −250 ms and +200 ms relative to the time of peak speed. IBI postural drift was defined as the pitch difference between the end of one bout and the start of the next, representing the passive postural drift between consecutive swim bouts. Postural compensation residual was calculated by the addition of IBI postural drift and the subsequent bout rotation. Residual variability was defined as the median absolute deviation of the compensation residual.

Navigation metrics were computed from epochs containing multiple swim bouts. Displacement per second was calculated using epochs comprising five consecutive bouts, defined as the Euclidean distance between the starting positions of the first and fifth bouts, divided by the time elapsed between them. Directional change was defined as the absolute change in swim direction between two consecutive bouts [26]. The absolute directional changes between bouts 1 and 5 were summed and divided by elapsed time to obtain the directional change per second.

To visualize circadian effects, we extracted data from 10 a.m. on the first day (7 dpf) to 10 a.m. on the third day (9 dpf). For each experimental repeat, kinematic parameters were binned into 2-hour intervals, and the median was calculated for each bin. For comparisons between day and night, we excluded data from the transition periods, defined as the hour before and after the day-night boundary: 8 to 10 a.m. for the night-to-day transition, and 10 p.m. to 12 a.m. for the day-to-night transition. To calculate standardized values for circadian plots, we performed z-transformations for each experimental repeat. Autocorrelation analyses of fluctuation periods were also conducted per experimental repeat, with cross-correlation coefficients normalized to the value at lag 0.

### Statistical analysis

We used a 3 × 2 factorial design to test the effects of three photoperiodic treatments (LL, DD, LD) and two circadian phases (day and night) on zebrafish behavior. For each behavioral parameter, we assessed its distribution to select the appropriate statistical tests. None of the parameters followed a normal distribution, so we proceeded with non-parametric methods for all analyses.

In summary tables, results from all experimental repeats were pooled, and parameter distributions were reported as medians with interquartile ranges (IQR). P-values were calculated using the median test. Effect sizes for median tests were estimated as $\sqrt{\chi^{2}/N}$, a transformation commonly used to express chi-squared statistics as a standardized effect size analogous to correlation coefficients [34]. Scott’s rule was applied to determine the number of bins for all histograms [35].

For comparisons reported in the main manuscript, each experimental repeat was summarized by its median, and the means of these medians were compared across conditions using independent *t*-tests. *P*-values and Cohen’s *d* were reported in the main text and figure legends.

Correlations of bout rotation and IBI drift were examined using bi-square regression, a robust method that reduces sensitivity to extreme values. Statistical analyses of correlation coefficients were performed using two-way ANOVA with post-hoc Tukey HSD tests with the Bonferroni correction applied to control for Type I errors.

### Data and code availability

All raw data and code for analysis have been uploaded to the Open Science Framework: 10.17605/OSF.IO/36TFU.

## RESULTS

### Larvae employ distinct swimming kinematics under dark and light

**Figure 1:**
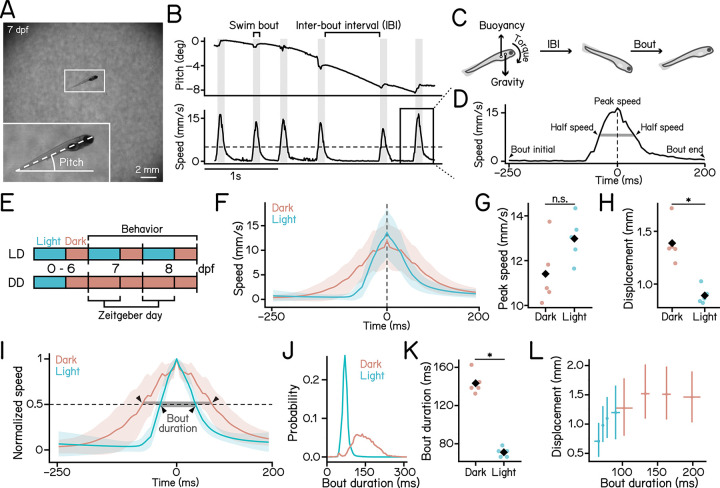
Measuring posture and locomotion in freely swimming zebrafish larvae reveals distinct kinematics in dark and light. **(A)** Representative image of a 7 day-post-fertilization (dpf) zebrafish larva recorded in the SAMPL apparatus. Pitch angle is defined as the angle of the trunk axis (dashed line) relative to the horizontal. Positive values indicate a nose-up posture. **(B)** Example epoch containing multiple swim bouts, with pitch angle (top) and swim speed (bottom) plotted over time. The horizontal dashed line marks the 5 mm/s threshold used for bout detection. Gray vertical bars indicate the duration of swim bouts. The inter-bout interval (IBI) is defined as the time between two bouts. **(C)** Schematic illustrating the forces acting on a larva along the vertical axis. Because the center of mass lies anterior to the center of buoyancy, larvae experience a nose-down rotation during IBIs and correct their posture through swim bouts. **(D)** Time series of swim speed during a swim bout. The vertical dashed line indicates the time of peak speed. Bout duration (gray bar) is defined as the duration between the two half-speed points (arrowheads). **(E)** Schematic illustration of experimental paradigm. Larvae were raised under a standard 14–10 light-dark cycle from 0 to 6 dpf and transferred into the SAMPL apparatus for behavioral recording from 7 to 9 dpf under either light-dark (LD) cycle or constant-dark (DD) conditions. Data from the zeitgeber day were used for analysis. **(F)** Swim speed plotted as a function of time under dark (red) and light (cyan) conditions. Solid lines represent the mean across 5 experimental repeats; shaded areas indicate ±1 standard deviation (SD) across repeats. **(G)** Peak swim speed under dark (red) and light (cyan). Median values for each experimental repeat are plotted as dots. Black diamonds indicate group means (p = 8.053e-02, Cohen’s d = 1.265, t-test). **(H)** Bout displacement under dark (red) and light (cyan). Median values for each experimental repeat are plotted as dots. Black diamonds indicate group means (p = 7.831e-04, Cohen’s d = 3.314, t-test). **(I)** Time series of normalized swim speed. For each bout, speed is normalized to its peak value. Solid lines represent the mean across 5 experimental repeats; shaded areas indicate ±1 SD. Bout duration is defined as the duration (gray bar) between the two time points where speed is at 50% of the peak value (arrowheads). **(J)** Histogram of bout duration under dark (red) and light (cyan) conditions. **(K)** bout duration under dark (red) and light (cyan) conditions. Median values for each experimental repeat are plotted as dots. Black diamonds indicate group means (p = 1.315e-06, Cohen’s d = 8.090, t-test). **(L)** Swim bout displacement plotted as a function of bout duration. For each condition, data were grouped into four equal-sized quartiles based on bout duration. Crossing points indicate the median of displacement and bout duration of each quartile. Horizontal and vertical error bars represent the interquartile ranges (IQRs) within each group for bout duration and displacement, respectively. n = 23875/98722 bouts from 105/98 fish for dark/light over 5 experimental repeats. See also Table 1.

We first examined how lighting affects kinematics of freely swimming zebrafish larvae. We recorded larval activity using a custom-designed Scalable Apparatus to Measure Posture and Locomotion (SAMPL) [25]. SAMPL records larvae from their side (Figure 1A) and measures swim speed and posture on the pitch (nose-up/nose-down) axis (Figure 1B) at a frame rate of 166 Hz [25]. Zebrafish larvae swim in short, discrete bouts interspersed with inactive periods called inter-bout intervals (IBI) (Figure 1B). Due to their anteriorly positioned center of gravity relative to the center of buoyancy (Figure 1C), larvae are inherently unstable and rotate nose-down during IBIs [14](Figures 1B and 1C). We recorded swim parameters from 7 to 9 days post-fertilization (dpf) and extracted a 450 ms window of activity for each bout (Figure 1D). We generated light-dark (LD) and dark-dark (DD) photoperiodic conditions by turning the daylight LED in the apparatus on or off during the zeitgeber day, repectively, and compiled day-time data for analysis (Figure 1E).

Compared to siblings under the light, larvae in the dark reached comparable peak speed (Figures 1F and 1G; dark: 11.409 ± 1.445 mm/s vs. light: 12.982 ± 1.003 mm/s, *p* = 8.053e-02). However, we observed greater swim displacement in the dark (Figure 1H; dark: 1.388 ± 0.195 mm vs. light: 0.891 ± 0.082 mm, *p* = 7.831e-04). We found that dark bouts exhibited more gradual changes in speed (Figure 1F). To quantify the dynamics of the speed profile, we normalized each swim bout by its peak speed and measured the bout duration, defined as the duration during which larvae moved at speeds greater than half their peak speed (Figure 1I). Larvae in dark showed significantly longer bout durations compared to their siblings in light (Figures 1J and 1K; dark: 143.373 ± 11.588 ms vs. light: 71.084 ± 5.040 ms, *p* = 1.315e-06).

These findings led us to hypothesize that larvae achieve greater displacement through longer bouts. We plotted displacement as a function of bout duration and observed a positive correlation in the light condition (Figure 1L, cyan), indicating that increased bout duration is associated with greater displacement. However, this relationship was absent in bouts recorded in the dark (Figure 1L, red), suggesting that larvae employ a different locomotor regime without light, and leaving open the question of the functional significance of extending bout duration in the dark.

These results demonstrate that zebrafish larvae exhibit distinct swim kinematics in light versus dark conditions, and that *bout duration* reliably differentiates activity between these conditions.

### Swim bouts in the dark stabilize posture

Zebrafish larvae rotate in the pitch axis during swim bouts [14, 25, 26]. Longer bout durations allow more time for rotation. Given the significantly longer bout durations in the dark, we hypothesized that larvae use these extended swim bouts to achieve greater body rotation.

**Figure 2:**
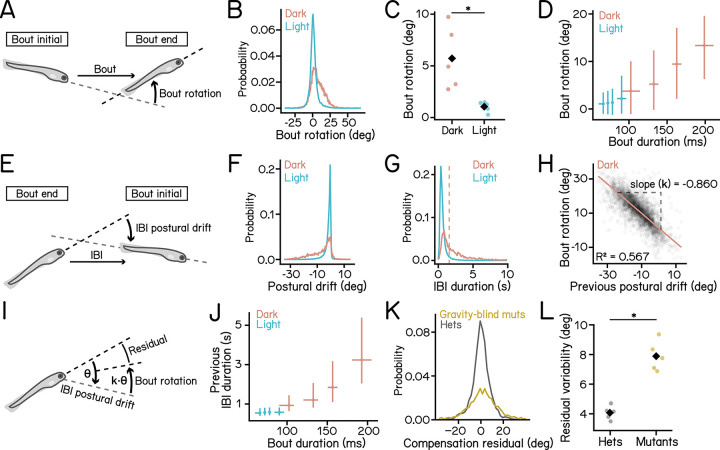
Swim strategy in dark compensates for postural drifts accrued during prolonged inactivity. **(A)** Schematic illustration of bout rotations, defined as the change in pitch angle during a swim bout. **(B)** Histogram of bout rotation under dark (red) and light (cyan) conditions. **(C)** Bout rotation under dark (red) and light (cyan) conditions. Medians for each experimental repeat are plotted as dots. Black diamonds indicate group means (p = 9.538e-03, Cohen’s d = 2.142, t-test). **(D)** Bout rotation plotted as a function of bout duration. For each condition, data were divided into four equal-sized quartiles based on bout duration. Crossing points indicate the median rotation and bout duration of each quartile. The horizontal and vertical error bars represent IQRs. **(E)** Schematic illustration of IBI postural drift, defined as the change in the pitch angle from the end of the previous bout to the beginning of the current bout. **(F)** Histogram of postural drift under dark (red) and light (cyan) conditions. **(G)** Histogram of postural drift under dark (red) and light (cyan) conditions. The dashed red line indicates the median IBI duration in the dark condition. Bouts with durations longer than this median are classified as long IBIs. **(H)** Scatter plot of bout rotation following long IBIs vs. previous IBI postural drift in dark. The red line represents the robust bi-square regression fit (slope k = −0.860, coefficient of determination *R*^2^ = 0.567) **(I)** Schematic illustration of compensation for postural drift following long IBIs. Larvae rotate nose-up proportionally to the amount of nose-down drift during the previous IBI. Compensation residual is defined as the sum of IBI postural drift and Bout rotation. **(J)** Previous IBI duration plotted as a function of bout duration. For each condition, data were divided into four equal-sized quartiles based on bout duration. The horizontal and vertical error bars represent IQRs. Crossing points indicate median of duration and bout duration of each quartile. **(K)** Histogram of compensation residual of gravity-blind *otog*^−/−^ mutants (yellow) and heterozygous control (gray). Results from bouts in the dark with long proceding IBIs were plotted. **(L)** Residual variability of gravity-blind *otog*^−/−^ mutants (yellow) and heterozygous control (gray). Medians for each experimental repeat are plotted as dots. Black diamonds indicate group means (p = 5.652e-05, Cohen’s d = 4.881, t-test). n = 23875/98722 bouts from 105/98 fish for dark/light over 5 experimental repeats. n = 3143/34285 middle bouts from 3-bout sequences were selected for calculation of IBI postural drifts for dark/light conditions. For the gravity-blind dataset, n = 3106/1716 bouts with long preceding IBIs from 99/136 fish for hets./mutant over 5 experimental repeats. See also Figure S1 and tables 1 and 2.

We measured rotations during swim bouts (Figure 2A) and observed significantly greater nose-up rotations in dark bouts (Figures 2B and 2C; dark: 5.724 ± 3.042°, light: 1.053 ± 0.504°, *p* = 9.538e-03). Notably, bout rotations were positively correlated with bout duration in the dark (Figure 2D, red), indicating that larvae extend their bout duration to achieve greater nose-up rotations.

Why do they rotate more nose-up in the dark? Because larvae experience a constant nose-down torque, we reasoned that in the dark they would accumulate larger nose-down postural drifts during inter-bout intervals (Figure 1C). We quantified kinematics during IBIs (Figure 2E) and found that larvae in the dark showed increased nose-down drifts (Figure 2F) and prolonged IBI durations (Figure 2G). These results support the hypothesis that larvae rotate nose-up to compensate for nose-down drifts accrued during prolonged IBIs in the dark.

To directly test this hypothesis, we examined correlations between IBI postural drift and subsequent bout rotation. We categorized swim bouts into those with long vs. short IBIs based on the median dark IBI duration (Figure 2G, dashed vertical line). We plotted bout rotations against IBI postural drifts during either the preceding or following IBI (Figure S1). Bout rotations in the dark were negatively correlated with postural drift during long IBIs, whereas no such correlation was observed under the light condition (Figures S1A to S1C). Interestingly, bout rotations were strongly linearly correlated with drift during the preceding IBIs (Figure 2H, *R*^2^ = 0.567), but not the following IBIs (Figure S1B, *R*^2^ = 0.172, quantified in Figure S1D), suggesting that larvae compensate for prior postural drift rather than anticipate future drift. To measure the degree of compensation, we defined the absolute slope of the best-fit line as the compensation gain (Figure 2H). Swim bouts in the dark exhibited a gain of 0.860(Figure 2H), indicating that following long IBIs larvae effectively compensate for nose-down rotations accrued during the IBI through swim bouts (Figure 2I). Correspondingly, bouts after longer IBIs in the dark exhibited longer bout durations (Figure 2J). These results demonstrate that, larvae perform nose-up counter rotations to compensate for nose-down drifts accrued during long periods of inactivity.

Building upon this observation, we further examined whether the IBI duration or the magnitude of postural drift that determines the amount of compensatory rotation. To dissociate IBI duration with the amount of passive postural drift, we took the advantage of a balance challenge that exacerbates the nose-down destabilizing torque. We used low-concentration copper sulfate to ablate the lateral-line hair cells, which reduced swim bladder size and resulted in increased nose-down angular velocity during inactivity [33] (Figure S1E). Hair-cell-lesioned larvae showed greater nose-down rotation during IBI despite having shorter IBI durations (Figures S1F and S1G), and rotated more nose-up during swim bouts (Figure S1H). Excitingly, we observed a similar linear relationship between bout rotation and postural drift during preceding IBIs (Figure S1I and table 2, *R*^2^ = 0.545) and measured a compensation gain of 0.756. These results demonstrate that the amount of compensatory rotation is not determined by the duration of the inactive period but instead by the amount of passive postural drift accrued.

Next, we asked whether the sensation of gravity contributes to the compensatory rotation. We used the *otogelin* mutant [36], which lacks the utricular otolith for the first two weeks of life (Figure S1J) and thus cannot sense gravity [37–39]. We examined bouts from gravity-blind larvae following long IBIs as they swam in darkness. To assess the effectiveness of compensatory rotation, we quantified the compensation residual, defined as the sum of IBI postural drift and the bout rotation (Figure 2I). Gravity-blind larvae showed a broader distribution of compensation residual than their heterozygous siblings (Figure 2K), reflected in significant higher residual variability in mutants (Figure 2L; heterozygous siblings: 4.070 ± 0.458°, mutants: 7.892 ± 1.008°, *p* = 5.652e-05). These results show that vestibular sensation via the inner-ear otolith is essential for effective postural compensation.

We conclude that larvae in the dark use long swim bouts with pronounced nose-up rotations to compensate for nose-down postural drifts during prolonged inactivity, reflecting a locomotor strategy to stabilize posture.

### Locomotor strategy in the light favors exploration

Previous studies reported that light increases activity level and swim distance of larval zebrafish [19, 40], thereby promoting effective exploration [41]. In contrast, we found that although larvae initiated swim bouts more frequently in the light (Figure 2G), individual bouts generated shorter displacements than those in the dark (Figure 2D). This discrepancy prompted us to investigate how larvae chained individual swim bouts to explore the water column effectively in the light.

**Figure 3:**
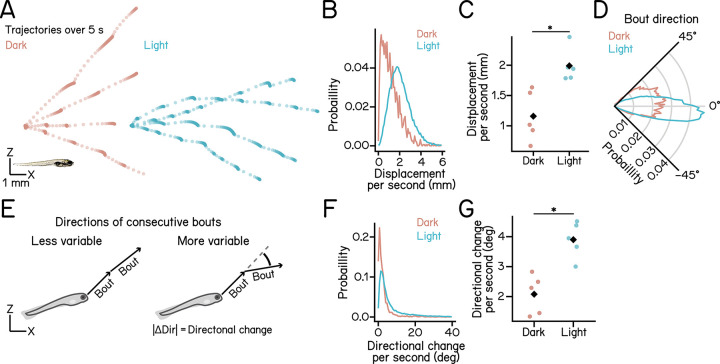
Swim bouts in the light travel further with greater directional variability per unit time. **(A)** Five-second trajectories of larvae under dark (red) and light (cyan) conditions. Four trajectories moving left to right are shown per condition. Dots indicate fish positions at 30 ms intervals. A 7 dpf larva is shown at the bottom left for scale. **(B)** Histogram of Euclidean displacement per second under dark (red) and light (cyan) conditions. **(C)** Per-second Euclidean displacement under dark (red) and light (cyan) conditions. Medians for each experimental repeat are plotted as dots. Black diamonds indicate group means (p = 5.882e-03, Cohen’s d = 2.352, t-test). **(D)** Polar histogram showing distributions of bout directions. **(E)** Schematics illustrating directional changes across bouts, defined as the sum of absolute changes in swim direction between consecutive bouts. Arrows indicate swim bout directions. **(F)** Histogram of directional change per second under dark (red) and light (cyan) conditions. **(G)** Per-second directional change under dark (red) and light (cyan) conditions. Medians for each experimental repeat are plotted as dots. Black diamonds indicate group means (p = 1.862e-03, Cohen’s d = 2.881, t-test). n = 7980/120010 sets of consecutive bouts from 105/98 fish for dark/light over 5 experimental repeats. n = 23875/98722 swim bouts for (D). See also Table 3.

We compared swim trajectories between light and dark conditions and observed more swim bouts and overall longer distance traveled in the light (Figure 3A). To quantify swim displacement efficiency, we calculated displacement per second by dividing the Euclidean displacement of the bout sequence by the total duration (refer to Methods). We found that larvae in the light traveled significantly farther than those in the dark over the same period (Figures 3B and 3C; dark: 2.317 ± 0.831 mm/s, light: 3.980 ± 0.556 mm/s, *p* = 5.882e-03). Larvae primarily swim horizontally in the light while performing more climbs in the dark (Figure 3D). These results demonstrate that larvae travel farther in the light by combining short individual swim bouts with faster swim rates.

The ability to change movement direction is essential for environmental exploration. Previously, we measured changes in swim directions on the vertical axis across consecutive bouts [26] and found that larvae in the dark swim with stable trajectories, enabling effective climbs and dives [26] (Figure 3E). Here, we examined how short, frequent swim bouts in the light influence changes of swim directions.

We first calculated the average change in direction between adjacent bouts. Bouts in the light showed slightly smaller directional changes than those in the dark (dark: 5.541 ± 0.701°, light: 4.123 ± 0.668°, *p* = 1.129e-02). We then assessed how directional changes accumulate over time. We calculated the rate of directional changes by dividing the total magnitude of change across a bout sequence by its duration (refer to Methods). Larvae in the light exhibited significantly greater directional changes per unit time than those in the dark (Figures 3F and 3G; dark: 2.080 ± 1.315°/s, light: 3.901 ± 1.211°/s, *p* = 1.862e-03).

These results led us to conclude that, under light conditions, larvae employ short, frequent bouts to achieve greater displacement and higher directional variability per unit time, reflecting a strategy that favors exploration.

### Dissociation of circadian and lighting effects on locomotor strategies

The circadian clock internally regulates locomotor activity. We hypothesized that kinematics of postural control and navigation are rhythmically modulated by the circadian clock.

**Figure 4:**
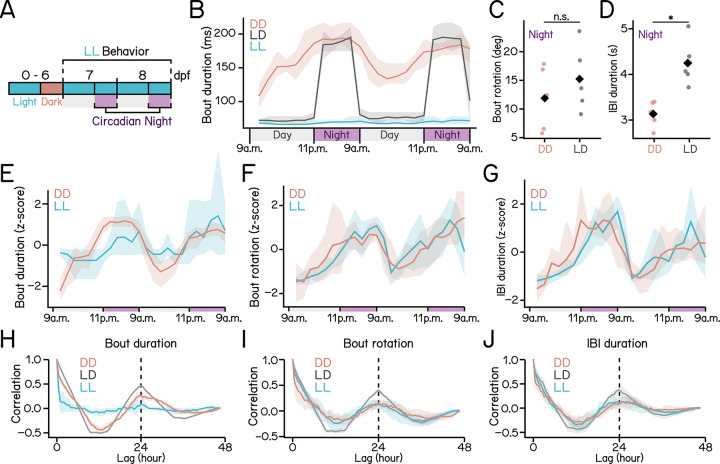
Dissociation of lighting and circadian effects on swim kinematics. **(A)** Experimental paradigm for the light-light (LL) condition. **(B)** bout duration plotted as a function of time over two days of recording under three lighting conditions: DD (red), LD (black), and LL (cyan). For each experimental repeat, a median bout duration within each 2-hour bin was calculated. Solid lines represent the means of medians. Shaded areas indicate the range of values across repeats. **(C)** Bout rotation during circadian night under DD (red) and LD (black) conditions. Median values for each experimental repeat are plotted as dots. Diamonds indicate group means (p = 3.813e-01, Cohen’s d = 0.586, t-test). **(D)** IBI duration during circadian night under DD (red) and LD (black) conditions. Medians for each experimental repeat are plotted as dots. Diamonds indicate group means (p = 2.792e-03, Cohen’s d = 2.689, t-test). **(E–G)** Z-transformed bout kinematics plotted as a function of time. Data were binned into 2-hour intervals and z-scored within each experimental repeat. bout duration **(E)**, bout rotation **(F)**, and IBI duration **(G)** are shown over two consecutive days. Solid lines represent the means of medians. Shaded areas indicate the range of values across repeats. **(H–J)** Autocorrelation of measured parameters over time. Data were binned into 0.5-hour intervals. Normalized autocorrelation scores of bout duration **(H)**, bout rotation **(I)**, and IBI duration **(J)** are plotted over lag in hours. Solid lines represent the means of medians. Shaded areas indicate ±1 standard deviation across repeats. Vertical dashed lines mark hour 24. n = 23875/15359/7618 DD bouts, 98722/11043/18019 LD bouts, and 100674/37586/27273 LL bouts for day/night/transition from 105/98/98 fish for DD/LD/LL over 5 repeats. See also Table 4.

We included a third photoperiodic condition with constant lighting (LL) (Figure 4A) and compared bout durations across DD, LD, and LL larvae over 48 hours of behavioral recording (Figure 4B). Larvae in constant darkness showed significant circadian rhythms, with bout duration increased further at night (Figure 4B, red). Strikingly, larvae under LD exhibited a pronounced biphasic pattern, with fast transitions at light-dark switches (Figure 4B, black). At night, bout duration and rotation were indistinguishable between DD and LD conditions (Figures 4B and 4C, rotation: DD: 11.892 ± 5.644°, LD: 15.248 ± 5.811°, *p* = 3.813e-01), whereas larvae in LD exhibited longer IBI durations (Figure 4D; DD: 3.134 ± 0.287 s, LD: 4.249 ± 0.511 s, *p* = 2.792e-03). In contrast, larvae in LL maintained short bout durations at night (Figure 4B, cyan), indicating a masking effect of light.

To better characterize circadian effects, we standardized kinematic parameters and examined their temporal dynamics (Figures 4E to 4G). Strong kinematic rhythms emerged in both DD and LL conditions, with larvae showing greater bout durations, increased nose-up rotations, and longer IBIs during circadian night (Figures 4E to 4G; quantified in Table 4). To visualize circadian periods, we performed autocorrelation analysis and observed correlation peaks at approximately 24 hours (Figures 4H to 4J). However, circadian rhythmicity of bout duration appeared muted under the LL condition (Figure 4H). We compared bout duration between day and night in the LL condition and found bout durations to be slightly longer at night (Table 4). Nevertheless, the difference was only 6 ms, corresponding to the temporal resolution limit of SAMPL [25].

Together, these findings show that light exerts a masking effect that promotes the exploratory strategy, while the circadian clock rhythmically modulates the magnitude of each strategy. Our results disentangle the respective contributions of endogenous rhythms and environmental lighting to the regulation of locomotor strategies in larval zebrafish.

## DISCUSSION

We defined two distinct locomotor strategies in larval zebrafish and demonstrated how environmental lighting and circadian rhythms collectively shaped postural and depth control. Under light conditions, larvae used frequent, short swim bouts with increased directional variability that promoted horizontal exploration. In the absence of light, larvae performed long bouts with greater nose-up rotations that compensated for postural drifts accrued during inactivity. While lighting had a dominant, masking influence on the locomotor strategy, circadian rhythms modulated the extent of each strategy. Our results demonstrate how fish adjust swim strategies to achieve longer periods of inactivity while maintaining postural and depth control. This work elucidates how environmental cues and the internal clock collectively determine locomotion strategies in a freely moving small vertebrate.

### Circadian and light modulation of balance and navigation

Circadian and lighting cues affect animal behavior [1–8, 42]. Traditionally, rhythmic activities were quantified using measures such as wheel running, beam crossings, and total distance traveled – metrics that primarily served as circadian phase markers [16, 17, 43, 44]. Recent advancements in machine-learning-based video analysis have provided powerful tools for extracting posture and movement kinematics in freely moving animals [45, 46]. However, their intensive computational demands hinder their use in extended recordings. Conversely, approaches using automatic classification enabled tracking of rhythmic behavior through multiple days [47, 48], but they often lack the resolution needed for detailed kinematic analysis in freely behaving animals. How changes in activity levels translate into kinematics that describe behavioral strategies remains largely unknown. We addressed these challenges by combining the simplicity of zebrafish behavior with high-resolution, real-time pose estimation [25]. We achieved multi-day behavioral datasets by recording larvae that stochastically entered a 20 mm × 20 mm field of view in the center of the arena [25]. This enabled us to define locomotor strategies in freely swimming fish and identify circadian regulation of postural control and locomotion.

Classic paradigms for zebrafish circadian studies use dim illumination ranging from 3 to 40 lux for the constant light condition [16, 17, 42, 49–52]. Larvae exposed to light intensities above this range were thought to be constantly active with a complete loss of circadian rhythm [52]. However, we used regular ambient light measured between 50 to 150 lux, yet we detected circadian modulation of swim kinematics under constant light. Our results indicate that circadian rhythms in larval zebrafish persist under constant moderate illumination. We attribute this discovery to the nature and resolution of our behavioral measurement. By recording from the side, we excluded larvae that were resting at the bottom of the chamber. Our high-resolution recording enabled detailed kinematic analysis, thereby enhancing our sensitivity to circadian fluctuations.

We observed a dominant effect of light on larval locomotor strategy, consistent with the classic ”masking effect” [5–7]. Light-induced masking of behavior has been reported across species, including mammals [8, 53, 54], birds [55], reptiles [56, 57], fish [18, 23, 24, 42, 52], and fruit flies [1, 58]. Nevertheless, mechanisms linking the modulation of sensory-motor circuits to the masking effect of light remain elusive. Unlike mammals, zebrafish can detect light directly via the pineal gland and in many other cell types [59–61]. Recent data indicate that both eyes and extra-retinal photosensitive tissues contribute to behavioral modulation in larval zebrafish [42, 62, 63]. Future studies combining loss-of-function approaches with deep phenotyping of light masking could elucidate the functional roles of different light-sensing pathways in locomotor behavior.

Our results revealed rhythmic fluctuations in postural control kinematics in LL and DD conditions, suggesting that balance behavior could be modulated by the time of day. This finding adds to the limited evidence of circadian influences on balance [64, 65]. Larval zebrafish are an emerging model for studying vestibular function and underlying circuits [14, 26, 32, 66–68]. Recent work focusing on the zebrafish optomotor response has identified mechanisms through which circadian time modulates circuit dynamics and behavior [69]. Future studies that integrate kinematic analysis of balance behavior with measurement of vestibular circuit activity will be essential for uncovering how circadian rhythms shape vestibular processing and balance control.

Together, our work sets the stage for exploring neural mechanisms by which lighting conditions and the internal clock collectively modulate motor outputs for postural control and locomotion in a small, diurnal vertebrate.

### Compensatory body rotation implies short-term memory of vestibular signals

Zebrafish are diurnal, showing prolonged periods of inactivity between swim bouts in the dark or at night. We found that larvae compensate for postural drifts by swimming with significant nose-up rotations. This strategy enables longer inactive periods while minimizing unfavorable postures. Unexpectedly, the amount of compensatory rotation was not associated with the duration of the inactive phase but was determined by the amount of passive postural drift. To achieve this, larvae might need to remember either their posture following a swim bout or postural drift during the inactive phase. These findings suggest the existence of a short-term memory of vestibular signals on the pitch axis.

Classic heading direction cells integrate angular velocity over time to encode directional and positional signals in the horizontal plane [70–74]. In complete darkness, vertebrates rely on vestibular input to maintain directional tuning when external reference points are unavailable [70]. Unlike horizontal rotation, where only angular velocity can be detected, tilt angles in vertical planes (pitch and roll) can be inferred from gravitational signals detected by the otolith organs in the inner ear [75]. In larval zebrafish, the semicircular canals remain nonfunctional during the period we studied here [76]. Nevertheless, the inner-ear otoliths enable sensation of gravity and linear acceleration [67, 77]. Evolutionarily conserved brainstem vestibular circuits transform otolithic input into motor outputs that control eye movements, stabilize posture, and maintain swim directions [26, 32, 38, 78, 79]. Our results demonstrate that larvae rely on otolithic signals to restore heading on the pitch axis following prolonged inactivity. Future studies may identify the neural substrates responsible for the storage of vestibular signals that enable postural compensation.

By characterizing the compensatory body rotation, our work lays the foundation for investigating neural mechanisms of vestibular processing using larval zebrafish.

### Limitations

The behavioral data acquired in this study are subject to several limitations arising from design choices and technical compromises of our apparatus.

First, the size of our behavior chamber raises questions about the ecological relevance of exploration in this context. Compared to previous studies that assessed exploratory swimming in 35 mm dishes holding 5 mL of water [41, 80], our setup contained 5 to 7 larvae swimming in 30 mL of water measured approximately 50.8 mm (L) × 50 mm (H) × 12.7 mm (W) [25]. Given that the volume of a 7 dpf larva, approximately 4 mm in length and 0.6 mm in width, can be estimated at around 0.4 to 0.8 mm^3^ [81], our setup is roughly equivalent to humans diving in an Olympic-sized swimming pool. A larva swimming continuously in the same direction would take 25–50 seconds to cross the chamber. Thus, we reason that we measured kinematic parameters of larvae exploring the environment. It is plausible that by introducing novel environments and points of interest, future studies will reveal more sophisticated exploratory strategies and enehance ecological validity.

Second, to achieve high-resolution imaging over 48 hours, we recorded larvae that stochastically entered a 20 mm × 20 mm field of view in the center of the arena [25] . While this approach enabled detailed analyses of locomotor kinematics, it was less suitable for measuring the free-running period than for capturing the global activity of all larvae. Moreover, although we observed fast adaptation of locomotor strategies to light transitions, we could not determine how quickly larvae adjusted their swim strategy during the light transition. Previous work revealed distinct acute responses to loss of illumination within 3 minutes [63, 82], suggesting that our recording likely missed acute responses but captured longer-term effects of changes in lighting. In addition, due to the limited field of view, we could not determine the percentage of time larvae swimming in the water column vs. staying at the bottom. Although they, on average, adopted 13° climbs in the dark – potentially compensating for sinking between bouts – larvae likely also rest for extended periods on the bottom of the arena. Future studies using a deeper arena with global activity tracking would help elucidate the full range of resting and sleep behaviors in fish.

Third, the 166 Hz acquisition frame rate of our apparatus [25] limited the temporal resolution of bout duration measurements, hindering our ability to detect subtle circadian fluctuations under constant light (Figure 4H). Higher frame-rate recordings would improve this resolution and enable quantification of additional kinematics with fast temporal dynamics, such as body bend and tail beat. These measurements will offer deeper insights into circadian modulation of motor control and the underlying neuronal circuits.

Lastly, because the white-light LED strips and their usage varied across apparatus, light-condition recordings were collected at luminance levels ranging from 50 to 150 lux. Future studies with precisely controlled illumination will reveal how light intensity influences postural control and navigation in larval zebrafish.

## Conclusion

We demonstrated that lighting and circadian cues shape distinct locomotor strategies in larval zebrafish, prioritizing postural control in the dark and environmental exploration in the light. Our findings highlight how the interplay of external and internal cues governs balance and navigation in a freely moving small vertebrate.

## Supplementary Material

Supplement 1

## Figures and Tables

**Table 1:** Kinematic parameters under DD, LD, and LL during zeitgeber day. Refer to Figures 1–2.

Parameter	Unit	DD day (dark)	LD day (light)	P-value	Effect size	LL day (light)

**Bout parameters**						
Peak speed	mm/s	11.342 [6.238]	13.200 [7.248]	<0.001	0.119	12.711 [6.739]
Displacement	mm	1.440 [0.932]	0.914 [0.711]	<0.001	0.243	0.825 [0.635]
Bout duration	ms	144.578 [54.217]	72.289 [12.048]	<0.001	0.494	66.265 [12.048]
Bout rotation	deg	7.532 [14.454]	1.329 [5.194]	<0.001	0.213	1.717 [5.605]

**Inter-bout interval parameters**						
IBI duration	s	1.584 [2.253]	0.554 [0.392]	<0.001	0.270	0.554 [0.422]
IBI rotation	deg	−4.267 [10.867]	−0.816 [2.038]	<0.001	0.173	−0.752 [2.325]

Condition	Unit	Median [IQR]	MAD			

**Compensation residual (bouts following long IBIs, DD day)**						
Wild type	deg	−0.514 [5.361]	2.660			
Hair cell ctrl	deg	−0.253 [5.657]	2.832			
Hair cell lesion	deg	−0.250 [6.704]	3.324			
*otog* hets	deg	0.194 [8.164]	4.079			
*otog* mutants	deg	1.651 [15.601]	7.862			

Data from 5 experimental repeats were pooled to calculate median [IQR]. n = 23875/98722/100674 day-time bouts from 105/98/98 fish for wild type DD/LD/LL. Median test p-values reported for DD day vs. LD day comparisons; Effect size estimated using standardized chi-squared statistics. n = 8336/2994 bouts from 114/114 fish for hair cell control/lesions. n = 3106/1716 bouts from 99/136 fish for *otog* hets/mutants. MAD: median absolute deviation. See Methods for details.

**Table 2:** Correlation of bout rotation and IBI rotation under dark and light. Refer to Figure 2.

		DD day (dark)			LD day (light)		
		
IBI length	Relation	Slope	*R* ^2^	Bootstrapped p-value	Slope	*R* ^2^	Bootstrapped p-value

**Wild type**							
Long	Previous IBI	−0.860	0.567	<0.001	−0.360	0.164	<0.001
	Next IBI	−0.874	0.172	<0.001	−0.648	0.088	<0.001
Short	Previous IBI	−0.986	0.185	<0.001	−0.753	0.034	<0.001
	Next IBI	−0.712	−0.093	<0.001	−0.282	0.011	<0.001

**Lateral-line hair cell lesion control**							
Long	Previous IBI	−0.852	0.451	<0.001			
Short	Previous IBI	−1.131	0.226	<0.001			
**Lateral-line hair cell lesioned**							
Long	Previous IBI	−0.756	0.545	<0.001			
Short	Previous IBI	−1.004	0.371	<0.001			

Wild-type data from 5 experimental repeats were pooled and analyzed using bi-square regression. Middle bouts from 3-bout sequences were selected for analysis. n = 3143/34285 day-time bouts from 105/98 fish for DD/LD over 5 experimental repeats. The hair-cell lesion dataset contains 8336/2994 day-time bouts from 114/114 fish for control/lesions over 5 experimental repeats.

**Table 3:** Swim displacement and directional changes of larvae during zeitgeber day. Refer to Figure 3.

Parameter	Unit	DD day (dark)	LD day (light)	P-value	Effect size	LL day (light)

Displacement per second	mm	1.019 [1.117]	1.977 [1.258]	1.067e-154	0.166	1.897 [1.270]
Bout displacement	mm	1.440 [0.932]	0.914 [0.711]	<0.001	0.243	0.825 [0.635]
Bout direction	deg	12.971 [30.907]	−0.095 [19.875]	<0.001	0.183	0.828 [20.144]
Directional change per second	deg	1.959 [2.391]	4.005 [6.197]	1.318e-129	0.151	3.800 [5.775]
Directional change between bouts	deg	4.809 [8.064]	3.792 [7.043]	4.199e-49	0.041	3.648 [6.552]

Data from 5 experimental repeats were pooled to calculate median [IQR]. P-values for DD vs. LD comparisons are from the median test. n = 23875/98722/100674 day-time bouts from 105/98/98 fish for DD/LD/LL for individual bout displacement. n = 7980/120010/132090 sets of 5 consecutive bouts from 105/98/98 fish for DD/LD/LL for average displacement and directional change calculation. Median test p-values reported for DD day vs. LD day comparisons; Effect size estimated using standardized chi-squared statistics. See Methods for details.

**Table 4:** Circadian effects on kinematic parameters under DD and LL. Refer to Figure 4.

Parameter	Unit	DD				LL			
	
		Day	Night	P-value	Effect size	Day	Night	P-value	Effect size

**Inter-bout intervals**									
IBI time	s	1.584 [2.253]	3.145 [3.283]	<0.001	0.305	0.554 [0.422]	1.157 [1.518]	<0.001	0.287
IBI rotation	deg	−4.267 [10.867]	−13.459 [15.301]	<0.001	0.310	−0.752 [2.325]	−3.822 [8.252]	<0.001	0.230

**Bouts**									
Peak speed	mm/s	11.342 [6.238]	9.243 [4.900]	<0.001	0.200	12.711 [6.739]	11.115 [6.622]	<0.001	0.111
Displacement	mm	1.440 [0.932]	1.341 [1.056]	7.831e-26	0.053	0.825 [0.635]	0.712 [0.628]	2.173e-210	0.083
Bout duration	ms	144.578 [54.217]	180.723 [60.241]	<0.001	0.325	66.265 [12.048]	72.289 [12.048]	<0.001	0.128
Bout rotation	deg	7.532 [14.454]	14.000 [15.826]	<0.001	0.201	1.717 [5.605]	6.002 [9.556]	<0.001	0.247

Data from 5 experimental repeats were pooled to calculate median [IQR]. P-values for day vs. night comparisons are from the median test. We excluded data during the day-night transition period (8–10 a.m and 10 p.m.–0 a.m., see Methods). n = 23875/15359/7618 bouts and 100674/37586/27273 bouts for DD and LL during day/night/transition time. Median test p-values reported for day vs. night comparisons; Effect size estimated using standardized chi-squared statistics. See Methods for details.
