# Supplementary material for: Lighting and circadian cues shape locomotor strategies for balance and navigation in larval zebrafish
Source: bioRxiv. 2025 Nov 18:2025.11.18.689084. Preprint. [Version 1] doi: 10.1101/2025.11.18.689084 (PMC12667949; doi:10.1101/2025.11.18.689084)
Supplement: Supplement 1 [file NIHPP2025.11.18.689084v1-supplement-1.pdf]

bioRxiv preprint doi: https://doi.org/10.1101/2025.11.18.689084; this version posted November 18, 2025. The copyright holder for this preprint (which was not certified by peer review) is the author/funder, who has granted bioRxiv a license to display the preprint in perpetuity. It is made available under aCC-BY 4.0 International license.

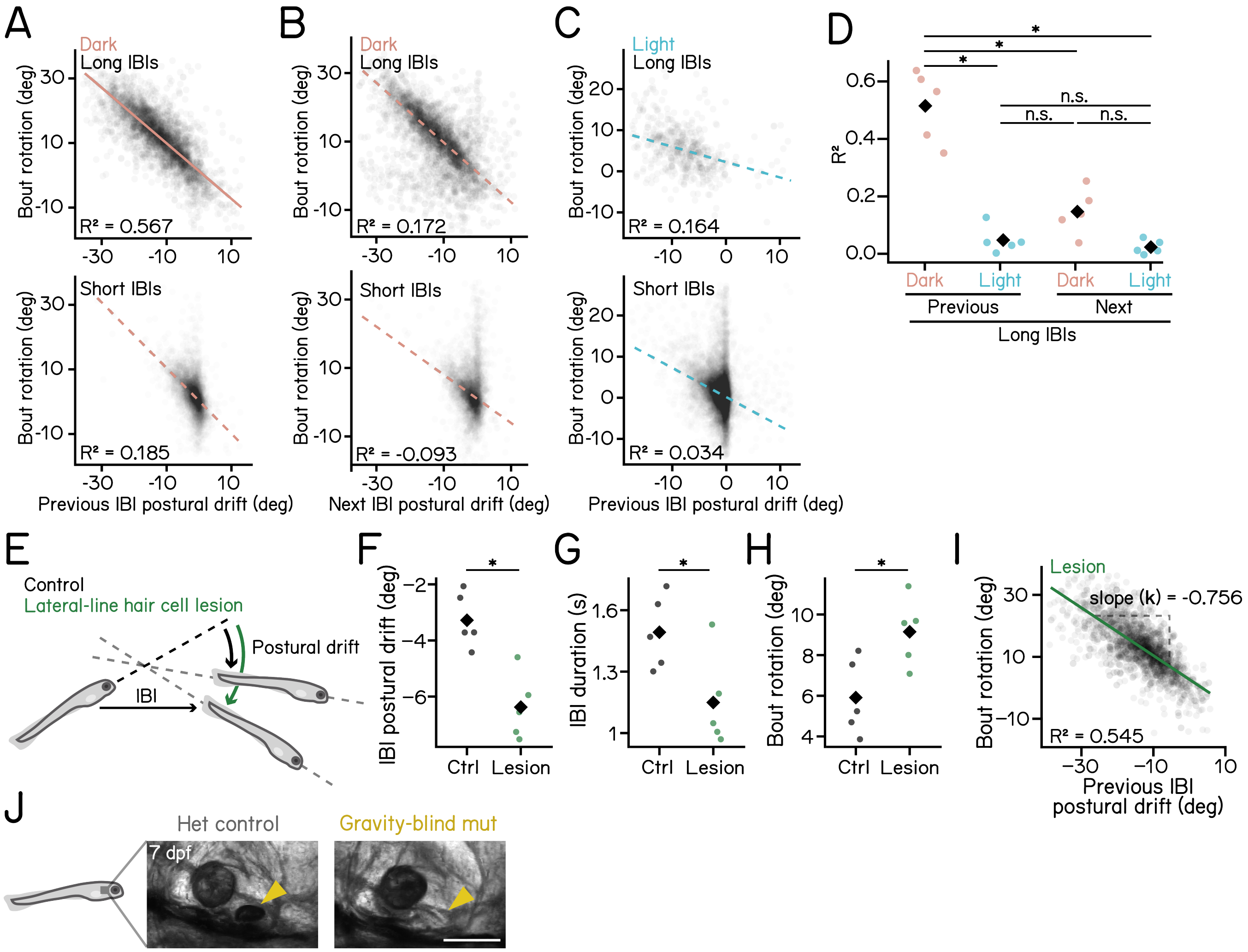

Figure S1: Bout rotations following long swim intervals correlate with IBI postural drifts. Refer to Figure 2.

(A) Scatter plot of bout rotation vs. postural drift during the previous IBI in the dark. Bouts were categorized into long and short IBIs based on the duration of preceding IBIs. Red lines represent the robust bi-square regression fit. Slope of the best-fit line for long IBIs: -0.860, R^2 = 0.567; slope for short IBIs: -0.986, R^2 = 0.185.

(B) Scatter plot of bout rotation vs. postural drift during the following IBI in the dark. Bouts were categorized into long and short IBIs based on the duration of following IBIs. Red lines represent the robust bi-square regression fit. Slope of the best-fit line for long IBIs: -0.874, R^2 = 0.172; slope for short IBIs: -0.712, R^2 = -0.093.

(C) Scatter plot of bout rotation vs. postural drift during the previous IBI in the light. Bouts were categorized into long and short IBIs based on the duration of preceding IBIs. Cyan lines represent the robust bi-square regression fit. Slope of the best-fit line for long IBIs: -0.360, R^2 = 0.164; short IBIs: -0.753, and R^2 = 0.034.

(D) Comparison of R^2 values for regression fits of bout rotation against IBI postural drift (*: adjusted p < 0.001, two-way ANOVA with post-hoc Tukey HSD tests).

(E) Schematics illustrating effects of lateral-line hair cell lesions on IBI postural drifts.

(F–H) Comparisons of bout kinematics between lateral-line lesioned larvae and sham controls: IBI rotation (p = 1.882e-03, Cohen's d = 2.876, t-test) **(F)**, IBI duration (p = 2.992e-02, Cohen's d = 1.667, t-test) **(G)**, and bout rotation (p = 2.028e-02, Cohen's d = 1.826, t-test) **(H)**. Median values for each experimental repeat are plotted as dots. Diamonds indicate group means.

(I) Scatter plot of bout rotation vs. drift during the previous IBI following lesions. Only bouts with long preceding IBI were plotted. The green line represents the robust bi-square regression fit. Slope of the best-fit line for long IBIs: -0.756, coefficient of determination R^2 = 0.545.

(J) *otog* mutants lack the utricular otolith (arrowheads) at 7 dpf. Scale bar: 100 µm.

Middle bouts from 3-bout sequences were selected for IBI postural drift computation. n = 3143/34285 day-time bouts from 105/98 fish over 5 experimental repeats for wild type DD/LD. For (F–H), n = 25008/8982 day-time bouts from 114/114 fish over 5 experimental repeats for lateral-line hair cell control/lesions. For (I), n = 2994 middle bouts from 3-bout. See also Table 2.
